# Understanding the phenotypic variability in Niemann-Pick disease type C (NPC): a need for precision medicine

**DOI:** 10.1038/s41525-023-00365-w

**Published:** 2023-08-11

**Authors:** Macarena Las Heras, Benjamín Szenfeld, Rami A. Ballout, Emanuele Buratti, Silvana Zanlungo, Andrea Dardis, Andrés D. Klein

**Affiliations:** 1 Centro de Genética y Genómica, Facultad de Medicina, Clínica Alemana Universidad del Desarrollo, Santiago, 7780272 Chile; 2 Department of Pediatrics, University of Texas Southwestern (UTSW) Medical Center and Children’s Health, Dallas, TX 75235 USA; 3 Molecular Pathology Group, International Centre for Genetic Engineering and Biotechnology (ICGEB), Trieste, 34149 Italy; 4 Departamento de Gastroenterología, Facultad de Medicina, Pontificia Universidad Católica de Chile, Santiago, 8330033 Chile; 5 Regional Coordinator Centre for Rare Diseases, University Hospital of Udine, 33100 Udine, Italy

**Keywords:** Molecular medicine, Personalized medicine

## Abstract

Niemann-Pick type C (NPC) disease is a lysosomal storage disease (LSD) characterized by the buildup of endo-lysosomal cholesterol and glycosphingolipids due to loss of function mutations in the *NPC1* and *NPC2* genes. NPC patients can present with a broad phenotypic spectrum, with differences at the age of onset, rate of progression, severity, organs involved, effects on the central nervous system, and even response to pharmacological treatments. This article reviews the phenotypic variation of NPC and discusses its possible causes, such as the remaining function of the defective protein, modifier genes, sex, environmental cues, and splicing factors, among others. We propose that these factors should be considered when designing or repurposing treatments for this disease. Despite its seeming complexity, this proposition is not far-fetched, considering the expanding interest in precision medicine and easier access to multi-omics technologies.

## Introduction

Lysosomal storage diseases (LSDs) comprise a family of over 70 monogenic disorders. Most LSDs have an autosomal recessive pattern of inheritance. They are caused by defective lysosomal proteins, including soluble acidic hydrolases, integral membrane proteins, lipid and ion transporters, enzyme modifiers, activators, or non-lysosomal proteins crucial for lysosomal function. This results in the progressive accumulation of various metabolites within lysosomes, interfering with their integrity and function^1,2^. These rare disorders are generally classified according to the age of onset of the clinical symptoms^1,3^. Furthermore, in many LSDs, secondary storage of substrates unrelated to the primary genomic defect can result from defects in non-enzymatic lysosomal proteins, but the mechanisms are poorly understood. Regardless of the mechanism, continuous substrate accumulation within lysosomes can initiate cascades of cytotoxic effects, leading to cellular damage, cell death, and eventually, organ dysfunction and degeneration, especially in the central nervous system (CNS)^4^.

In LSDs, the genotype cannot entirely predict the severity of the phenotype or the clinical course. In fact, LSDs are notable for being highly heterogeneous despite having an eventually progressive course with varying degrees of neurological involvement. As such, the clinical phenotypes of LSDs are regarded to fall somewhere on a continuum spectrum that ranges from mild to severe or rapidly progressive forms^3^.

The biological factors accounting for the broad phenotypic spectrum of LSDs, including the different ages of symptoms onset, rates of disease progressions, severity, degrees of organ involvement, and response to the pharmacological treatments, remain poorly studied^5,6^.

## Niemann-Pick disease type C (NPC): causes, subtypes, and clinical manifestations

NPC disease is a progressive neurovisceral autosomal recessive LSD caused by loss of function variants in the *NPC1* or *NPC2* genes^7,8^. *NPC1* is located on chromosome 18q11-q12, spanning 56 kilobases (kb), and contains 25 exons. *NPC2* is located on chromosome 14q24.3, spans 13,5 kb, and has five exons^8^. Most NPC patients (up to 95%) have mutations in *NPC1*^9^, with the remaining few (less than 5%) in *NPC2*.

For a long time, NPC was reported to have an incidence of about 1/100,000–1/120,000 live births^10–12^. However, in recent years, several teams have suggested that such an incidence is a significant underestimation of its actual incidence. For instance, using next-generation sequencing data from four independent projects, Wassif and colleagues have estimated an incidence of the juvenile or classical forms of NPC of ~1:90,000, and even a higher estimated incidence of 1:19,000–36,000 for the late-onset phenotype (i.e., adult) subtype^13^.

NPC1 is a highly glycosylated protein of 1278 amino acids located in late endosomes and lysosomes (LE/Lys). It has 13 transmembrane domains (TMD), five of them (TMD 3–7) form the sterol detection domain (SSD), which is highly conserved within a subclass of membrane proteins that participate in cholesterol metabolism^14–16^. NPC1 also contains three luminal domains that play a role in protein-protein interactions: the C-terminal domain, the middle luminal domain, and the N-terminal domain, which is highly conserved and has a leucine-zipper motif. The N-terminal tail binds to the 3β-hydroxyl end of the cholesterol molecule with nanomolar affinity^17,18^. NPC2 is a cholesterol-binding glycoprotein of 132 amino acids also located in LE/Lys^19^. NPC2 recognizes the iso-octyl side chain of the cholesterol molecule and exposes the 3β-hydroxyl end to NPC1 for its export outside these organelles^18,20^. Historically, Winkler et al.^21^ used the Saccharomyces cerevisiae NPC system (composed of NCR1, the NPC1 equivalent, and NPC2 proteins) as a model to greatly contribute to the understanding of how sterols can be transferred through the lysosomal membrane: first, sterols would be transferred between the hydrophobic pockets of vacuolar NPC2 and the membrane protein NCR1. Second, sterols would pass through a tunnel formed in the central core of NCR1, bypassing the glycocalyx that covers the luminal side of the vacuolar membrane and mediating the insertion of the sterol into the membrane^21^.

In addition to cholesterol, other lipids, particularly glycolipids, have been shown to accumulate in several tissues^22,23^. Cholesterol and sphingomyelin are the major lipids stored in peripheral tissues. GM2 and GM3 gangliosides are observed in NPC brains, although glucosylceramide, lactosylceramide, and gangliotriaosylceramide are also known to build up in this organ. The accumulation of unesterified cholesterol in peripheral tissues (mainly in the liver and the spleen) is associated with the infiltration of activated macrophages, which leads to the production of proinflammatory cytokines and plays a critical role in cell death^24,25^. The buildup of gangliosides in the CNS causes structural and functional damage to neurons (formation of mega neurites, extensive growth of ectopic dendrites, formation of neurofibril tangles, neuroinflammation, and dystrophy), particularly in the cerebellar Purkinje neurons, triggering the characteristic motor dysfunction observed in the disease^26,27^. The dysfunction of non-neuronal cells in the brain, such as microglia and astrocytes, also contributes to neurodegeneration^9,28–31^.

**Fig. 1:**
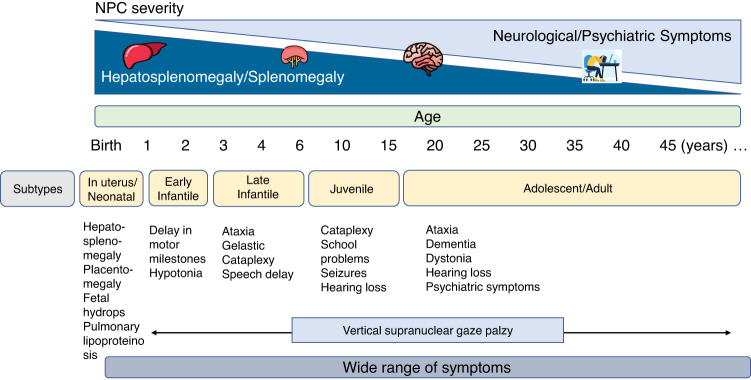
Wide range of symptoms and severity of NPC. The main differences are in the degrees of hepatosplenomegaly, splenomegaly, and in neurological and psychiatric symptoms. This diagram shows the great phenotypic variation in NPC disease and within each subtype of it. Adapted from Vanier 2015^31^.

Clinical presentations of NPC disease are highly heterogeneous and vary from a rapidly progressing neonatal form to an adult-onset chronic neurodegenerative condition. The life expectancy of the patients ranges from a few days until over 60 years of age, but most patients survive to 10–25 years of age^32^, although probably this is an underestimation According to data collected from a large cohort of French NPC patients, the age of onset of neurological symptoms predicts disease severity and determines life expectancy but current data suggests that NPC should be considered a continuum spectrum (Fig. 1, adapted from ref. ^33^). NPC is usually classified into four general categories according to the age of onset of neurological symptoms as follows^33–35^:

## Visceral-neurodegenerative forms

### In utero and neonatal (newborn <3 months)

In this form of disease, the internal organs are the most affected^30^. Patients can present in-utero splenomegaly, in-utero hepatomegaly, in-utero ascites, and intrauterine growth retardation^36^. Placentomegaly has also been reported^36^. After birth, symptoms include congenital thrombocytopenia, congenital anemia, and petechial rash. Some patients present with fetal hydrops or fetal ascites^30^. Others suffer from a prolonged neonatal cholestatic icterus, associated with progressive hepatosplenomegaly^37,38^. Pulmonary alveolar lipoproteinosis has also been described^39,40^.

## Early-infantile (3 months to <2 years)

Hepatosplenomegaly and/or prolonged neonatal jaundice are usually observed; however, hepatosplenomegaly can be the only symptom^37,38^. Other less frequent infantile forms with neurological involvement appearing by 12–18 months have been described^41,42^. This latest form is characterized by hypotonia and delay in developmental motor milestones, usually the first neurological symptoms. In these children, visceral disease is also generally present. The subsequent clinical course involves a loss of acquired motor skills, cognitive regression, and pronounced spasticity. In addition, patients may present with tremors, vertical supranuclear gaze palsy (VSGP), and seizures. Many children never learn to walk, and most die before age five.

## Neurodegenerative forms (Classical form)

These are the most common forms of the disease, which correspond to ~60–70% of all cases. They are classified into two subgroups: the late-infantile and juvenile onsets. Patients with a late-infantile onset and intractable epilepsy tend to die the earliest. Nonetheless, many live until their teens, but others can survive into their twenties or thirties^30^.

Neurological symptoms, including ataxia, dysmetria, dysarthria, and dysphagia, among others, are associated with neurodegeneration, especially in the cerebellum, due to extensive and progressive Purkinje and cortical neuronal death, followed by hippocampal and autonomic nuclei dysfunction^30^.

## Late-infantile (2–6 years)

These patients can also present with isolated hepatosplenomegaly or splenomegaly. The first neurological symptoms include gelastic cataplexy (frequent) and hearing loss, which becomes evident in the adult forms of the disease^35^. Patients can show clumsiness due to ataxia, gait disturbance, frequent falls, VSGP (vertical supranuclear gaze palsy, usually present but often unrecognized), fine motor skill impairments, speech delay, and epilepsy. As patients age, motor problems worsen, and they develop dysphagia, dysarthria, and dementia. Most patients with this form survive between 7 to 12 years of age^35^.

## Juvenile (6–15 years)

This is the second most frequent form of the disease. The first sign is splenomegaly and, less commonly, hepatosplenomegaly, which also may be detected earlier (including the neonatal period). These patients suffer a significant motor impairment and a variable cognitive decline. They may manifest poor school performance, language and learning difficulties, impaired attention, coordination problems such as clumsiness, frequent falls, progressive ataxia, dystonia, and VSGP (often as the initial sign). Later, they may present with dysarthria, dysphagia, seizures (in about half of these patients), dystonic postures, and psychotic signs. Life expectancy is variable, but usually, they survive till their teens to young adulthood^30,35^.

## Psychiatric-neurodegenerative forms

### Adolescent/Adult (>15 years)

Adolescent and adult-onset NPC patients may represent up to a third of all NPC patients. This group may encompass a broad array of time to symptom onset/rate of disease progression. Neurological manifestations frequently occur in these patients. The typical presentation is a history of severe progressive ataxia, dystonia, VSGP, variable cognitive decline, and psychotic symptoms. Other commonly reported symptoms are dysarthria and dysphagia, which occur later. Patients with this form are less likely to present with seizures, gelastic cataplexy, and usually show mild visceral disease, which may have been known for years^30,35^.

Lately, large numbers of older NPC adults (>30 years) are being identified. Many of them are experiencing a relatively favorable quality of life. They have been able to complete school or be in professions as they present very slow disease progression, which usually starts with physical difficulties and memory impairment. They generally present with progressive hearing loss^35^.

The psychiatric symptoms include paranoid delusions, auditory or visual hallucinations, and interpretative thoughts. Other mental manifestations include depression, behavioral problems with aggressiveness, and social isolation. Several cases have also been reported with bipolar disorder and obsessive-compulsive^30,43,44^. Patients are frequently being treated for bipolar, schizophrenia, or psychosis ahead of the NPC diagnosis.

## Diagnosis

The combination of biochemical and genetic studies can confirm NPC diagnosis, and the analysis of biomarkers should be preferred as a first-line test to screen for NPC. Currently, three biochemical markers can be used in combination: oxysterols (cholesterol oxidation products) and lyso-sphingomyelin^45,46^. However, in all cases, the diagnosis must be confirmed by genetic analysis and, if necessary, by a Filipin test, a fluorescent probe that binds free cholesterol in skin fibroblasts^46^.

## Genotype-phenotype correlations in NPC disease

More than 500 disease-causing variants have been described in different regions of the *NPC1* and *NPC2* genes. It has been challenging to establish genotype–phenotype correlations, mainly due to the private nature of most *NPC1* variants and because even the most common variants (I1061T, P1007A, and G992W) are found in low frequency. However, some studies have shown a correlation between carrying nonsense or frameshift variants and a more severe neurological course. In addition, homozygous variants in SSD are usually deleterious. Variants in the cysteine-rich luminal loop, where the three most frequent variants are found, show a variable clinical impact. Still, they seem to have a minor effect on cellular trafficking. Concerning *NPC2*, on the other hand, a relatively frequent nonsense variant (E20X) and many different variants that lead to a truncated protein have been associated with very severe clinical phenotypes. In addition, it has been seen that the missense variants led to more varied phenotypes. These have been reported in juvenile and adult-onset forms of the disease^30,47,48^.

Dardis and colleagues^49^ performed a multicentric study to characterize the molecular basis of NPC in Italy. In this study, 105 patients belonging to 83 unrelated families were included. Seventy-seven families presented variants in the *NPC1* gene, while six in *NPC2*. The data obtained were consistent with previous reports: the mutational spectrum was highly heterogeneous, even in patients with the same causal variant and within the same family. Most patients with the early infantile severe lethal and the severe infantile forms were associated with the presence of null variants in both alleles (partial deletions, frameshift/nonsense variants, or splicing variants showed to be severe by functional analysis), while all patients affected by the juvenile and adult forms harbored missense variants in at least one allele^49^.

Unfortunately, the correlation between *NPC1* mutations and clinical phenotypes has not yet been thoroughly investigated at the molecular level. Recently, Shammas studied the pathogenicity of common *NPC1* gene variants by evaluating their effect on protein and cellular levels. They expressed NPC1-Flag tagged chimeras with and without variants in COS-1 cells and identified the biosynthetic forms of wild-type NPC1 by immunoprecipitation and endoglycosidase H (endo H) treatment. They found that mutant proteins in different domains of NPC1 had different trafficking patterns, with mutants that were partially trafficked or blocked in the ER associated with infantile clinical onset, late infantile, or juvenile-onset. However, the limited sample of mutants and the use of heterologous cellular models represent the limitations of this study^50^. Despite its limitations, however, this study established the concept of phenotypic heterogeneity of NPC1 variants at the biochemical and cellular levels and correlated trafficking classes along the secretory pathway of NPC1 variants with clinical phenotypes and symptom severity. This offers a potential method to assess the pathogenicity of different NPC1 mutants^50^.

Using the described methodology, the biochemical phenotypes of cultured fibroblasts from two NPC patients carrying different compound heterozygous *NPC1* variants were assessed^51^. The fibroblasts from patient one had a delay in trafficking along the secretory pathway, while fibroblasts from patient two had normal trafficking. The lipid levels were also measured in the fibroblasts, and patient one had higher cholesterol levels and lower levels of one type of glycolipid compared to patient two. Sphingosine levels were higher in both NPC fibroblasts, but phospholipid levels were not significantly different from normal cells. The distribution of a protein called flotilin-2, a classical lipid raft marker, was found to be variant-dependent. Treatment with Miglustat, a drug used to treat NPC, reduced cholesterol, and glycolipid levels in both patients. However, it did not change phospholipid and sphingosine levels or was able to restore normal lipid raft structure in NPC cells. These findings help explain why treatment effectiveness with this drug varies among patients^51^.

## Family cases with different severities

Though some variants are frequently detected as associated with specific phenotypic presentations, many exceptions exist, even within the same family, suggesting that additional genetic and/or non-genetic factors could act as disease modifiers.

In 2009, a case of phenotypic heterogeneity in two non-identical siblings, a male, and a female, with the adult-onset form of NPC disease-carrying the same causing variant, was reported by a different group^52^. They presented differential schizophrenia-like psychosis and showed dimorphism in illness course, clinical, and biochemical parameters. The male sibling presented with psychosis at age 16 and cognitive and motor disturbance at age 25, whereas his older sister showed less severe biochemical, neuroimaging, and ocular motor parameters. She presented with a similar schizophrenia-like illness with an associated cognitive and motor disturbance at the age of 31 years. In this case, the disease onset, course, and response to treatment replicate the sexual dimorphism seen in schizophrenia and suggest an interaction between the neurobiology of the metabolic disorder of these siblings and the sex differences in neurodevelopment^53^. In this respect, it is interesting to note that the sexual dimorphism in schizophrenia, concerning the onset and course of the disease, has been recognized since Kraepelin first described this disorder^54^.

As a result, it has been observed that most men experience their first schizoid episode in their late teens and early twenties, while most women become affected in their mid- to late- 20s. Moreover, men generally have greater severity of disease and worse long-term outcomes. Finally, women typically respond to lower doses of antipsychotic medication, although they show a higher rate of affective troubles in psychotic episodes^53^. At the molecular level, estrogen has been implicated in many of these variables because it can exert a protective effect on the structures involved in schizophrenia and the consequences of oxidative stress and excitotoxicity through its regulation of growth factors and ion channel function^55^. In addition, increased myelination in temporal areas has been observed in females during adolescence, suggesting a pro-myelination effect of female sex steroids on white matter development^56^. As these siblings showed a significant sexual dimorphism in the schizophrenia-like-psychosis that developed as the main symptom of adult-onset NPC, their case raised the possibility that gender can indeed influence the effects of NPC on the CNS, which could have implications for illness course, and treatment response. However, there is still insufficient data on humans on the effect of sex on the biochemical and clinical parameters of NPC disease.

In 2014, Benussi and colleagues^57^ reported the first case of extensive phenotypic variability in monozygotic twins carrying the same causal homozygous variant (c.2662 C > T; p.P888S) in the *NPC1* gene. They were diagnosed at the age of 24. One of the twins presented with a history of obsessive-compulsive disorder and slowly progressive inferior limb clumsiness, dysphagia, dysarthria, and moderate splenomegaly. Neurological examination revealed a broad-based ataxic gait, limb dysmetria, downward vertical gaze palsy, brisk lower limb reflexes, and ankle clonus. The neuropsychological assessment revealed global cognitive deficits in multiple domains. He also presented intrusive recurrent, and persistent thoughts that caused significant stress and anxiety. The second twin showed mild obsessive-compulsive disorder without impact on daily life activities. The neurological and neuropsychological evaluation revealed mild neurological impairment without the involvement of cerebellar functions. Fibroblasts from both twins displayed similar filipin staining with significant intracellular accumulation of unesterified cholesterol and increased plasma cholestane-3b,5a,6b-triol^57^.

Other cases of NPC within familial contexts displaying varying symptomatology have been documented. In a consanguineous Turkish family, three cousins presented with hepatosplenomegaly and progressive neurodegeneration. Regrettably, some family members succumbed to similar complaints. Notably, vertical supranuclear gaze palsy was not consistently observed across all cases. Through molecular diagnosis, a homozygous c.1553+5 G > A intronic mutation in *NPC1* was identified, resulting in exon 9 skipping and the production of an aberrant NPC1 protein^58^.

Another case involved two siblings who harbored compound heterozygous variants in exon 13 of the *NPC1* gene (c.1955C>G, p.Ser652Trp and c.2107 T > A, p.Phe703Ile). While both individuals presented with nonspecific neurological symptoms, the age of onset differed between them. One sibling exhibited dyspraxia and motor clumsiness at the age of 7, whereas the other displayed a systemic manifestation characterized by hepatosplenomegaly noted at 2 months of age, followed by neurological symptoms emerging at 4 years, including speech disturbance^42^. Other cases of NPC siblings with discordant phenotypes have been reported^34,59,60^.

In conclusion, the presence of substantial phenotypic variability among familial NPC cases suggests a contribution from various factors such as epigenetic variations, post-zygotic mutagenesis, modifier genes, or environmental influences. To comprehensively comprehend the underlying molecular mechanisms, further research is warranted.

## NPC mouse models of different genetic backgrounds for modifier gene mapping of disease severity

The term “modifier gene” refers to a locus where DNA sequence variation changes the phenotypes typically linked to “target genes” that act independently. The defining feature of modifier genetics is the interactions between specific alleles of the modifier and target genes^61^. Variations in modifier genes and pathways may explain why some patients with the same *NPC1* mutation exhibit heterogeneous phenotypes and disease progression.

Variants that inactivate *NPC1* have been described in other species, including mice. Two of the most widely used and well-characterized murine models of *Npc1* disease, BALB/c npc^nih^ ^62^ and C57BL/6J^spm^ (sphingomyelinosis or spm)^63^, arose by spontaneous mutation and led to a severe phenotype. However, recent reports describe other spontaneous or targeted alleles able to affect mice and induce an NPC phenotype. These include three alleles leading to a severe phenotype *Npc1*^*pf*^, *Npc1*^*imagine*^, and *Npc1*^*pioneer*^ ^64,65^; two leading less severe phenotypes, *Npc1*^*nmf164*^ and *Npc1*^*I1061T*^ ^66,67^; and one that allows tissue-specific deletion of *Npc1*, the *Npc1*^*flox*^ ^68^.

In 1986, ref. ^69^ investigated whether the expression of the spm might be influenced by the genetic background. C57BL/KsJ^spm/spm^ mice show striking hepatosplenomegaly with a prominent accumulation of sphingomyelin and cholesterol due to a deficiency of sphingomyelinase. In this study, they constructed two congenic strains, C57BL/6J^spm^ (selected as a related substrain) and DBA/2 J^spm^ (selected as a strain of different origin). The degree of hepatic sphingomyelinase deficiency in these three mutant backgrounds was similar. Specifically, in C57BL/6J^spm^ and DBA/2J^spm^ mice, hepatosplenomegaly was not pronounced despite increased levels of hepatic lipids. Nonetheless, the lifespan of C57BL/6J^spm/spm^ and DBA/2J^spm/spm^ mice was shorter than that of C57BL/KsJ^spm/spm^ mice whilst the increase in hepatic lipid levels was faster in C57BL/6J^spm/spm^ and DBA/2J^spm/spm^ mice than in C57BL/KsJ^spm/spm^ mice, which could be associated with the presence or absence of hepatosplenomegaly and shortened lifespan. In general, the occurrence of hepatosplenomegaly appeared to have a protective effect against the accumulation of lipids. Finally, histological studies showed the formation of massive foam cell clusters of variable size in the liver and spleen of C57BL/KsJ^spm/spm^ mice, whereas, in the case of the C57BL/6J^spm/spm^ and DBA/2J^spm/spm^ mice, foam cells were diffusely distributed in smaller clusters of relatively uniform size. Taken together, these findings suggest that the appearance of hepatosplenomegaly in mutant mice depends on the genetic background^69^.

More recently, a study aimed at identifying modifier genes affecting the age of onset of neurological signs was performed^70^ by linkage analysis by crossing the *Npc1*^*nih/+*^ mutant on the BALB/cJ background with DBA/2J mice. Then, the F1 progeny were intercrossed, and 540 F2 mice were generated, of which 88 were affected mice (*Npc1*^*nih/nih*^). The onset of neurological symptoms, defined as when a continuous extensor tremor of the forelimbs occurs for more than 6–7 s, was used as a trait to perform QTL analysis. The age range of the disease onset of the 88 affected F2 mice was from 39 to 62 days. Of these 88 affected mice, 22 were identified as more severely affected (13 female, 9 male; onset less than 47 days), and 23 mice were mildly affected (6 female, 17 male; onset past 54 days). When their genome was sequenced, researchers found a significant peak on chromosome 19 (LOD score = 2.24) around D19Mit88 that accounted for 52% of the variance observed for the neurological onset in the affected F2 progeny (unfortunately, they did not have enough power resolution to identify specific gene variants)^70^.

In parallel to these studies, Parra and colleagues^71^ have also investigated the role of genetic background on NPC progression. The authors transferred the *Npc1*^*nih*^ mutation from BALB/c to C57BL/6J genetic background by successive backcrossing with the offspring. The survival curves showed a striking difference between genetic backgrounds. *BALB/c-Npc1*^−*/*^^−^ mice died between 70 and 85 days, while *C57BL/6J- Npc1*^−*/*^^−^ mice died between 28 and 35 days. Interestingly, *BALB/ c-Npc1*^−/−^ mice gained weight until 6–7 weeks old and then started to lose weight whilst *C57BL/6J-Npc1*^−/*−*^ mice began to lose weight at 2–3 weeks old and were significantly smaller than *BALB/c-Npc1*^−^/^−^ mice at all ages. As NPC disease is a lipidosis that affects multiple organs, the authors measured the lipid content of the brain, liver, and spleen of 4-week-old mice. Accordingly, they observed that liver and spleen cholesterol content were increased in *Npc1*^*−*^*/*^*−*^ mice from both genetic backgrounds compared to the wild type. However, the relative increase in C57BL6/J-*Npc1*^*−*^*/*^*−*^ mice was more considerable. In addition, they evaluated the brain, liver, and spleen phospholipid content. They found that *BALB/c-Npc1*^*−*^*/*^*−*^ and *C57BL/6J- Npc1*^*−*^*/*^*−*^ mice showed no differences in brain or liver phospholipid content but observed a fivefold difference in the spleen of *C57BL/6J-Npc1*^*−*/−^ mice versus the wild type, which suggests that spleen damage due to lipid accumulation could be responsible for the phenotypic differences between these strains. Notably, they observed that the 4 weeks old brains and livers from both genetic backgrounds showed similar features. However, the spleens from *C57BL/6J- Npc1*^−^/^−^ mice showed severe histological and morphological abnormalities compared to their controls. Accordingly, *Npc1*^−^/^−^ mice from the C57BL/6 J genetic background showed significant spleen damage at early ages, whereas *Npc1*^−^/^−^ mice from the BALB/c genetic background showed minor spleen alterations^71^. Finally, no Purkinje cell degeneration was observed at this age in both genetic backgrounds. Although no specific genes were mapped, these results indicate that genetic background can strongly influence NPC disease severity.

Finally, in 2020 Rodriguez-Gil and colleagues^72^ assessed the impact of genetic background on NPC disease severity in mice. In their approach, they used CRISPR/Cas9 to generate a new NPC1 mouse mutant, *Npc1*^*em1Pav/em1Pav*^ (*Npc1*^*em*^), which has an in-frame deletion allele in the cysteine-rich domain of NPC1. These animals were generated and maintained on a C57BL/6J background and had many phenotypic features of NPC1 disease, such as reduced levels of NPC1 protein, lipid storage abnormalities (including accumulation of GSLs within visceral and neuronal tissue), visceral pathology (with the presence of foam cells in both the liver and spleen), Purkinje neuron loss in the cerebellum, motor impairment, neurodegeneration, and a reduced lifespan^72^. They then used marker-assisted selection/speed congenic techniques to establish a congenic strain of the Npc1^em^ allele on a BALB/cJ genetic background. This experimental design allowed the production of Npc1^em/em^ mice that showed ≥92% BALB/cJ homozygosity for all tested genetic markers by the N4 generation. When they analyzed these mutants at the N4 and N6 generations, the authors found that the new mice presented a significantly increased lifespan and less severe visceral pathology compared to the original C57BL/6J background. Analysis of N2 mice generated from a backcross using C57BL/6J and BALB/cJ found that Npc1^em/em^ mutants also had an increased lifespan with greater variance, which suggests that strain-specific modifiers influenced disease severity and survival. This study included QTL analysis for the lifespan of 202 N2 mutants on a mixed C57BL/6J and BALB/cJ background and found markers associated with significant LOD scores on chromosome 1 (LOD = 5.57) and chromosome 7 (LOD = 8.91)^72^. Most importantly, these regions will provide candidate genes for future study as modifiers that could contribute to the variable phenotypes observed in NPC1 patients.

In conclusion, several NPC mouse models have been described in different genetic backgrounds. With the current advances in genetics and genomics, it is expected that they will represent very valuable resources for dissecting the genomic architecture underlying disease severity and progression of NPC^73^. Once identified, it is hoped that these modifier genes will facilitate the development of severity biomarkers and customized therapeutic approaches for subsets of patients.

## Severity biomarkers

LSDs result in the expansion of the LE/Lys compartment in cells. Te Vruchte and colleagues^74^ showed that the relative lysosomal volume could serve as a potential biomarker for the age of onset and severity of these disorders. The assay uses the fluorescent probe Lysotracker, which accumulates selectively in the acidic compartments (LE/Lys). For their experiments, they stained B cells because they are a uniform population and do not change in response to infection. The assay was validated using resting splenic and circulating B cells from the NPC mice, and a progressive increase in relative acidic compartment volume was confirmed. Importantly, this expansion was seen at all ages (compared with age-matched controls) and was highly significant at each time point (3, 6, and 9 weeks) in both splenic and circulating B cells. Storage of GSLs in peripheral B cells occurred in parallel with the progressive storage of gangliosides and lactosylceramide in the forebrain of 2, 4, 7, and 9-week-old *Npc1*^*–/–*^ mice compared with WT controls. This indicates that the lipid storage rate in peripheral B cells can be used as a proxy for the storage in the CNS^74^. Following these results, the B-cell lysotracker assay was validated and expanded to more than 100 NPC probands derived from multiple independent clinical cohorts, and this approach confirmed that this biomarker correlated with age-adjusted clinical severity scores and the levels of oxysterols, so these data support the use of Lysotracker for monitoring NPC disease^75^.

Previous reports have shown that the age of onset of neurological symptoms correlates with life expectancy and disease progression in NPC1 patients^30,76^. Therefore, knowing the age of onset of neurological symptoms in patients is crucial to predict the course of the disease and thus identify candidates who might benefit the most from earlier treatments. In this respect, Baxter and colleagues showed that the LysoTracker levels in skin fibroblasts of NPC patients could be used as a predictor for the age of onset and disease severity^75^. Their study found that LysoTracker levels were inversely correlated with the age of onset and the age of the first neurological symptom (the highest correlation). In addition, LysoTracker levels were directly associated with neurological severity scores. In the search for specific transcripts levels that may serve as biomarkers of severity, these researchers performed RNA-Seq in 41 NPC1 primary fibroblasts untreated and treated with 2-Hydroxypropyl-β-cyclodextrin (HPβCD) for 24 h. The gene expression data were correlated with the three clinical parameters: age of onset, age of first neurological symptoms, age-adjusted neurological severity score, and LysoTracker staining. The expression levels of 127 genes correlated with at least one clinical or cellular phenotype: ten genes were associated with the age of onset, 40 with the age of first neurological symptom, 43 with neurological severity score, 21 with LysoTracker levels in untreated cells, and 27 with changed in LysoTracker levels following HPβCD treatment. Most genes did not overlap among these correlations (only 14 genes correlated with two phenotypes), suggesting that phenotypes’ severity is affected by several genes/pathways rather than a small set of genes. Nonetheless, it is important to highlight that the pathways enriched in the total of 127 genes identified with this approach coincided with the role of NPC1 protein in cholesterol and GSL homeostasis.

Most importantly, Ingenuity Pathway Analysis identified 7 of the 127 genes as highly relevant biomarkers, which can be detected in human blood: GPNMB, HBEGF, HMGCR, PIK3CA, RSF1, STK11, and TLR4. Moreover, the drug–gene interaction database identified drug targets for several of the 127 genes, with ~53% of them that could be considered strong candidates for drug treatments. At present, treatments with these drugs are still in their infancy. All that can be said at present is that HPβCD treatment (ΔHPβCD values) did not change the gene expression of the mRNA biomarkers, suggesting that HPβCD did not affect many potentially clinically relevant genes in B cells. This study was the first to collect patient transcriptomic data, correlate it with clinical severity and cellular phenotypes, and suggest potential drug candidates for clusters of NPC patients. Variations in modifier genes and pathways could explain why some patients with the same NPC1 causal variant exhibit heterogeneous phenotypes and disease progression^75^.

## Current therapeutic approaches and others under development

The primary molecular mechanism responsible for NPC symptomatology is the intra-lysosomal buildup of cholesterol and GSLs. Therefore, most current therapeutic interventions aim to lower their presence, either by inhibiting their synthesis or reducing their accumulation. Some drugs, such as Miglustat^77^, cyclodextrins (intravenous and intrathecally)^78–80^, and Vorinostat^80^, are the first drugs that have been tested in clinical trials for NPC. Still, several others have also been proposed, such as lovastatin, rapamycin, and arimoclomol, among others.

## Miglustat

Miglustat (*N*-butyldeoxynojirimycin; NB-DNJ; Zavesca®), is a blood–brain barrier (BBB) permeable small iminosugar that acts as a competitive inhibitor of the glucosylceramide synthase enzyme, which catalyzes the first step in glycosphingolipid synthesis^77^.

Miglustat treatments in NPC cats reduced neuronal GSL accumulation, and the cellular pathology in the brain slowed the neurological progression of the disease and increased lifespan^81^. These animal models were followed by initial human clinical trials. Encouragingly, Miglustat-treated patients showed neurological improvements or stabilization^82–84^, although frequently reported adverse events included diarrhea, flatulence, weight loss, and tremors^85^.

The European Medicines Agency (EMA) and the Food and Drug Administration (FDA) had already approved Miglustat for the treatment of Gaucher disease^86^. In 2009, EMA extended Miglustat’s indication to treat the progressive neurological manifestations in NPC patients^87^. This extension was followed by other countries such as Japan and Canada. At the moment, this drug is the only treatment approved for NPC disease; however, the FDA has not approved its use for NPC disease^88^.

## 2-Hydroxypropyl-β-cyclodextrin (HPβCD)

HPβCD is a cyclic oligosaccharide with a hydrophobic center. HPβCD transports free cholesterol from the LE/Lys compartments to the cytosol, which reduces its accumulation^24,89^.

Either systemic or direct administration of HPβCD into the CNS of *Npc1*^−/−^ mice normalized the biochemical abnormalities, reversed lysosomal cholesterol transport disturbances, increased their lifespans, significantly improved liver dysfunction, reduced lipid accumulation, and neurodegeneration^24,90^.

Clinical trials in NPC patients with NPC1 disease (NCT01747135, ClinicalTrials.gov) suggested that intrathecal treatment with HPβCD slowed disease progression^91^. Furthermore, a phase 1 trial involving intravenous administration of HPβCD demonstrated its biological activity in both the central nervous system and peripheral tissues of adult subjects^79^. The trial is registered under NCT02939547, and an open-label extension (NCT03893071), as well as a global pivotal trial (NCT04860960), are ongoing. The aforementioned phase 1 trial in patients was conducted subsequent to an extensive report on compassionate use of intravenous and intrathecal HPβCD, which concluded that the addition of intrathecal administration did not yield additional benefits, particularly considering the high doses used and the associated risks of neurotoxicities, including permanent and acute hearing loss^91^. In summary, these trials provide support for initiating longer-term clinical trials to assess the safety and efficacy of intravenous HPβCD in individuals with NPC^92^.

## Vorinostat

Another approach explored as a potential treatment for NPC patients involves using histone deacetylase (HDAC) inhibitors. One of them, Vorinostat, is an FDA-approved drug for treating cutaneous T-cell lymphoma^93^.

In preliminary experiments, Munkacsi and colleagues administered Vorinostat intraperitoneally for 5 days at a 150 mg/kg body weight dose into the *Npc1*^*nih/nih*^ mice^93^. Gene expression, liver function and pathology, serum and tissue lipid levels, body weight, and lifespan were used to follow disease progression. Studies showed a reversal of liver dysfunction. However, no positive effects on disease progression and animal survival were observed. Nonetheless, the metabolism of apolipoprotein B and the expression of the main components of lipid homeostasis in primary hepatocytes from mutant mice were altered by treatment with Vorinostat^93^. As a result, these results suggested that HDAC inhibitors could be applicable to the treatment of visceral NPC disease, but more work will be required to envision a future clinical trial with this compound.

In another similar study, Pipalia and colleagues treated NPC human fibroblasts with small HDAC inhibitors, which dramatically corrected the NPC phenotype in cells containing one or two copies of the NPC1 I1061T variant^94^. This effect was associated with increased NPC1 expression. Interestingly, the inhibition of HDACs was also identified as a candidate therapy for NPC in yeast^92^. The potential use of HDAC inhibitors for NPC treatment is high because they can cross the BBB and are being tested for mood behaviors^95^ in phase III clinical trials for several types of cancer^96^.

## Arimoclomol

Arimoclomol is a hydroxamic acid that amplifies heat shock protein (HSP) gene expression to enhance the endogenous cytoprotective mechanisms in the presence of cellular stress^97^. This drug helps to preserve cellular function and to prevent cell death in cells experiencing lysosomal stress^98^. Arimoclomol can cross the BBB, and it can be detected in the cerebrospinal fluid of amyotrophic lateral sclerosis-treated patients^98^.

Regarding NPC disease, Arimoclomol-treated *Npc1*^−/−^ mice re-activated the stress response in their brains, reducing neurodegeneration and improving locomotor coordination, but did not provide a rescue effect in the liver^99^. The treatment also enhanced myelination^100^. Furthermore, arimoclomol significantly reduced lysosomal storage and the buildup of unesterified cholesterol in NPC1 human fibroblasts^101^.

A Clinical trial showed that arimoclomol increased the likelihood of slowing disease progression^102^. Currently, the drug is available for its compassionate use (while collecting more safety data).

## Activation of the Ca^2+^-permeable endo-lysosomal two-pore channel 2 (TPC2) by small molecules

Lysosomal Ca^2+^ release has significant physiological relevance because it regulates several cellular processes, such as autophagy, membrane trafficking, exocytosis, nutrient adaptation, membrane repair, and cell migration^103^. It has been reported that lysosomal Ca2+ content or Ca2+ release disturbances are associated with several diseases, mainly LSDs^103,104^. Mucolipidosis type IV (MLIV) represents the most direct link between defective lysosomal Ca^2+^ liberation and neurodegeneration. This disease is caused by dysfunction of the lysosomal cation channel TRPML1^105^. However, TRPML1 signaling or TRPML1-mediated Ca^2+^ release is also impaired in other LSDs such as NPC1, Niemann-Pick type A (NPA), and Fabry disease^106^. Accordingly, it has been observed that TRPML1 activation in cells can rescue lysosomal storage phenotypes^106^. However, the effects of activating the related two-pore channel 2 (TPC2) in LSDs have been less explored to this date. Recently, Rosato and colleagues^107^ hypothesized that TPC2 activation could modulate Ca^2+^ signaling and rescue LSD phenotypes, mainly in LSDs where TRPML1 is affected. They showed that small molecule activation of TPC2 ameliorates some cellular phenotypes associated with LSDs, such as cholesterol or lipofuscin accumulation and abnormal vacuoles. These rescue effects were evaluated in MLIV, NPC1, and Batten disease patient fibroblasts and neurons derived from isogenic human iPSC models of MLIV and Batten disease. For the in vivo proof of concept, they also tested TPC2 activation in the MLIV mouse model, rescuing neuroinflammation, accumulation of P62/SQSTM1 inclusions, and improving motor behaviors. The data suggested that TPC2 is a promising target for treating different types of LSDs^107^.

## c-Abl inhibitors

The link between NPC neurodegeneration and the activation of the c-Abl/p73 apoptotic signaling has been studied in several disease models^108–111^. C-Abl is a kinase that phosphorylates and activates p73, a proapoptotic transcription factor. C-Abl and p73 are expressed in the cerebellum of NPC mice, and the levels of p73 target genes are increased. The expression of the active forms of p73 colocalizes with c-Abl and active caspase 3 in Purkinje cells. The inhibition of c-Abl with imatinib mesylate, an FDA-approved drug for treating chronic myelogenous leukemia, improves locomotor behaviors, reduces weight loss and neuronal apoptosis, and increases the survival of NPC mice [NO_PRINTED_FORM]. Thus, these inhibitors could be an excellent therapeutic alternative to attenuate the symptoms of NPC disease.

In parallel, it has been reported that histone deacetylation plays a relevant role in neuronal gene repression in neurodegenerative diseases^94^. In 2016, Contreras and colleagues^112^ demonstrated that c-Abl activity could also increase Histone deacetylase 2 (HDAC2) levels and activity, inducing neuronal gene repression in NPC models. As a result, c-Abl inhibition could prevent the increase of HDAC2 protein levels and activity in NPC neuronal models and the brain of mutant NPC mice^112^. Therefore, inhibition of c-Abl could be a pharmacological target for preventing the harmful effects of increased HDAC2 levels in NPC disease.

In addition to HDAC2, another report by Contreras and colleagues also explored the connection between c-Abl and the transcription factor EB (TFEB), a key element of lysosome signaling^113^. The study demonstrated that c-Abl phosphorylates TFEB on tyrosine and that its inhibition allowed TFEB translocation to the nucleus promoting the expression of its target genes. In addition, they found that c-Abl inhibitors promote a reduction in cholesterol accumulation in both in vitro and in vivo NPC models^113^. The TFEB-dependent clearance reveals the importance of the c-Abl/TFEB signaling as a therapeutic target for diseases with lysosomal dysfunction.

## Alternative cholesterol exit from the lysosome

In recent years, several studies have focused on bypassing deficiencies in NPC1 and favor the release of cholesterol from the lysosome through alternative pathways. Interestingly, lysosomes, as well as other organelles and cellular compartments, such as the plasma membrane, lysosomes, endoplasmic reticulum (ER), mitochondria, Golgi apparatus, and lipid droplets, physically interact and communicate with each other, preserving their compartmentalization through Membrane Contact Sites (MCSs)^114,115^. The opposition between two organelles can form MCS through tethering proteins and lipids that allow the transfer of metabolites, such as calcium and lipids, thus modulating the function of one or both compartments^114,115^.

As a result, MCSs between the ER and lysosomes are vital for maintaining cholesterol homeostasis. Interestingly, NPC1 and the ER-localized Gramd1b sterol-transporter are part of the ER-lysosome MCS^116^. Thus, when lysosomes accumulate cholesterol, Gramd1b contact with NPC1 allows the transfer of cholesterol to the ER. In cells with NPC1 deficiency, this ER-lysosome MCS is disrupted, contributing to cholesterol buildup in lysosomes^116^. In line with the principle, a therapeutic approach for NPC could therefore be the functional recovery of ER-lysosome MCSs. With this idea, Hoglinger et al., 2019^116^ overexpressed a lysosomal membrane ORP1L mutant that cannot sense or transport sterol but can serve as an artificial tether expanding ER-lysosome contacts through constitutive interaction with VAP on the ER. Accordingly, this approach induced a decrease in lysosomal cholesterol accumulation in NPC1-deficient Hela cells ^120^114]. Along the same lines, Meneses-Salas and collaborators found a recovery in the percentage of the lysosome surface in contact with the ER in CHO cells with mutations in the *Npc1* gene after silencing Annexin A6, which is involved in the regulation of cholesterol homeostasis by binding to membranes in a calcium-dependent manner.

These two studies suggest that it may be possible to postulate an increase of ER-lysosome MCSs as a potential strategy for new therapies for NPC disease^117^. Supporting this idea, the number of ER-lysosome MCSs was induced with agents that reduce cholesterol accumulation in NPC cells, such as hydroxypropyl- c-cyclodextrin (HPγCD) and hydroxypropyl-β-cyclodextrin (HPβCD) in NPC1 patient fibroblasts^118^.

## *N*-acetyl-l-leucine

*N*-acetyl-l-leucine, a modified amino acid, is used in the treatment of vertigo. Its mechanism of action involves the activation of cerebral glucose metabolism in the cerebellum and other regions of the brain^119,120^. Notably, *N*-acetyl-l-leucine has also shown promise in delaying disease progression and extending the lifespan of *Npc1*^−/−^ mice^121^.

Building upon these findings, an initial trial involving 12 NPC patients was conducted, wherein *N*-acetyl-l-leucine was administered for a duration of 1 month. The treatment resulted in improvements in ataxia, cognition, and overall quality of life for the patients^122^. Subsequently, a phase II clinical trial was carried out, demonstrating meaningful clinical improvements in symptoms, functioning, and quality of life after 6 weeks of treatment (NCT03759639)^123^.

Excitingly, a phase III randomized, double-blind, placebo-controlled, multi-center therapeutic study is currently underway (NCT05163288). This trial aims to evaluate the efficacy of *N*-acetyl-l-leucine in patients aged 4 and older, who have a confirmed diagnosis of NPC. Sixteen centers across Australia, the Czech Republic, Germany, the Netherlands, Slovakia, Switzerland, the UK, and the USA are participating in this study^124^. There is great anticipation surrounding the potential outcomes of this trial.

## One-size does not fit all. Need for personalized therapies

**Fig. 2:**
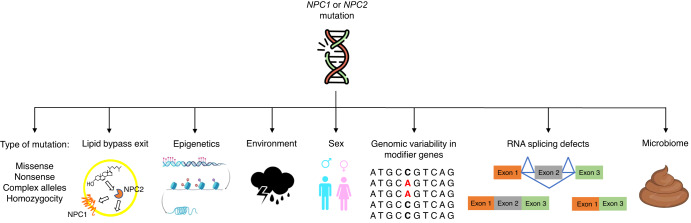
Potential contributing factors to the phenotypic variability in NPC. Some potential modifying variables include the type of mutation, activation of lipid bypass exit pathways, epigenetics, environment, sex, modifier genes, RNA splicing defects, and the microbiome (the latter has not yet been studied for any of the LSDs).

As already discussed, NPC disease symptoms are highly heterogeneous; however, one-size-fits-all potential therapies are usually proposed. Emerging data show that biological differences among individuals should be considered for designing therapeutic approaches (Fig. 2).

## Genomic variability in rapamycin response: a need for pharmacogenomics studies

Rapamycin activates autophagy and has immunosuppressive properties^125^. It is an FDA-approved drug and crosses the BBB^126^.

The effect of rapamycin in cellular models of NPC disease has shown contradictory results. On the one hand, autophagy induction with rapamycin increases cholesterol accumulation in human skin NPC cells, and its inhibition decreases, indicating that autophagy-derived cholesterol accumulates in NPC lysosomes^127^ Furthermore, NPC-induced mitochondrial fragmentation can be rescued by an autophagy inhibitor in human NPC neurons^128^. On the other hand, the induction of autophagy with rapamycin restored the autophagic flux in human NPC iPSC-derived hepatic and neuronal cells, increasing cell viability^129^. These data suggest that both inhibition and induction of autophagy could serve as therapeutic strategies for treating NPC disease.

Regarding this issue, Klein and Calderón tested the therapeutic outcomes of rapamycin on NPC disease progression in male *Npc1*^*−/−*^ mice from C57BL6/J and FVB/NJ genetic backgrounds^130^. Mice were injected with mock or rapamycin 10 mg/kg daily from postnatal day 20. The average lifespan of PBS-treated FVB/NJ-NPC mice was ∼75 days, while C57BL6/J-NPC lived ∼30 days. Interestingly in the C57BL6/J-NPC mice, treatment with rapamycin increased survival by 100%, whereas in FVB-NPC mice, rapamycin was toxic and reduced their life expectancy to ∼65 days.

In addition to the contradictory published cell-based data, this paradoxical result indicates that other factors besides loss of NPC1 function influence NPC pathophysiology and therefore how patients should be treated. To better understand these apparent contradictions, how rapamycin exerts its differential effects in NPC models must be further interrogated in the future.

## Other factors that could contribute to phenotypic variability in NPC

### Sex

Reports observing sex differences in survival times have been published in NPC mice. For example, Bianconi’s group^131^ showed that female *Npc1* mutant mice (BALB/c npc^nih^) live significantly longer (median survival of 78 days) than male *Npc1* mice (median survival of 66 days). In 2020, Cougnoux’s group^132^ reported similar data on the C57BL/6J background. The mean survival was 65 ± 7 days and 55 ± 16 for C57BL/6J female and male mutant mice, respectively. Bianconi’s group also used a survival dataset to determine whether survival differences existed between male and female NPC1 patients. Using this dataset, it resulted that the median survival time for males and females was 12 and 14 years, respectively^131^.

This characteristic also seems to be evolutionarily conserved because sex differences have also been reported in animal models of NPC. In addition, male NPC mice show a more significant weight loss and impairments in locomotor activity, coordination, and balance, but not in cognition, compared to females^133^. In another study, Holzmann and colleagues^134^ compared the therapeutic effects of Miglustat, HPßCD, allopregnanolone, and combined therapy (Miglustat, HPßCD, and allopregnanolone) on the brain, body weight, and behavior in a large cohort of BALB/c npc^nih^ mice, with emphasis on sex differences. All treatments in male and female mutant mice led to decreased loss of body weight and, partly, brain weight. Regarding motor coordination, assessed by the rotarod test, male mutant mice benefited from the combined treatment. In contrast, female mice benefited from the combined therapy and the unique Miglustat and HPßCD treatments. These results show that sex-dependent differences in response to therapies will become an important aspect to consider for treating patients in the best possible way.

### Environmental factors

As previously shown, the genetic background significantly influences the disease progression and survival of NPC1 mouse models^71,135^. However, other elements, such as environmental factors, could also modify disease progression and survival in NPC mouse models.

The first indication of this effect came from Liu and colleagues^135^ reported that two colonies of NPC mice (*Npc1*^*nih/nih*^) of the BALB/c genetic background that had been isolated from one another for 9 years showed different mean survival: one colony lived 89 ± 1.4 days, while the other lived 80 ± 0.8 days. The average survival previously reported for these mice ranges between 70 and 90 days^135^. On top of this, Cougnoux and colleagues^132^ reported that in their facility, the *Npc1*^*nih/nih*^ mice in the C57BL/6J genetic background lived 61 ± 12 days and had a predominantly neurological phenotype with loss of cerebellar Purkinje neurons in C57BL/6J *Npc1*^*nih/nih*^ mice, like that observed in *Npc1*^*nih/nih*^ BALB/c mice. However, as mentioned above, Parra and colleagues have reported that these mice lived 32 days^71^ and had a predominantly visceral phenotype.

Taken together, these results suggest that multiple environmental factors may explain some of these differences, such as pathogens in the facility, diet, accessibility to food, care or attention provided to animals, and genetic drift, among others. Considering some of the above observations and the fact that several reports have suggested that an acute immune response during pregnancy can affect brain development and have significant effects on the cerebellum^136^, Cougnoux’s group hypothesized that NPC disease pathology might be influenced by prenatal environmental factors, which could have long-term effects on the neuroinflammatory component of NPC pathology. This group evaluated the impact of maternal immune activation (MIA) on disease progression in an NPC1 mouse model^132^. To this end, they injected polyinosinic-polycytidylic acid (PolyI:C), an agonist of toll-like receptor 3 (TLR3) that mimics a viral, influenza-like infection, into 10- to 11-week-old Npc1^+/nih^ BALB/c pregnant mice when developing embryos were at embryonic day 9.5. In this experiment, they observed that PolyI:C-induced MIA triggered cerebellar manifestations, including increased expression of inflammatory biomarkers and decreased Purkinje neuron density^132^. These are hallmarks of NPC pathology, and therefore these researchers hypothesized that PolyI:C-treatments could alter *Npc1*^*nih/nih*^ disease progression. The results also supported that prenatal exposure to an acute immune response may worsen the motor function and the loss of Purkinje neurons (in lobules I/II and III) in female NPC mice without significantly affecting astrogliosis or microgliosis. In turn, these support previous observations showing that microglia in the *Npc1*^*nih*^ mouse model could have ambivalent functions (protective and deleterious)^25,27,137–139^. The small but significant loss of Purkinje neurons caused by MIA in female Npc1 mutant mice was reflected in the impaired motor functioning on the 24 mm wide balance beam. Unexpectedly, in male NPC mice, MIA did not seem to affect the Purkinje cell number, possibly because the loss of Purkinje neurons was already significant by the time it was measured. Thus, MIA appears to eliminate the female advantages observed in Npc1 mutant mice^132^.

In conclusion, although intrinsically difficult to study in a highly controlled manner, all the data obtained from these studies suggest that prenatal environmental exposures may be a contributing factor to the NPC phenotypic variability and future work in this direction would certainly be justified.

### mRNA processing defects

RNA processing involves several steps of maturation and quality control. Around 50% of the variants related to neurodegenerative diseases can affect pre-mRNA splicing^140,141^. For NPC, about half of the variants in the *NPC1* gene are nonsense or frameshift and can generate a premature stop codon. Aberrant transcripts can be degraded through mRNA nonsense-mediated decay (NMD); therefore, prematurely truncated proteins cannot be produced^142^. The NPC1 mRNA transcript does not seem resistant to this rule because degradation via NMD of NPC1 transcripts harboring premature stop codons generated by nine nonsense or frameshift variants and one deletion has been reported. As a result, the mRNAs and the protein were undetectable^143,144^. In addition to frameshift variants, splicing defects exist in NPC and many other LSDs, such as Fabry, Pompe, and mucopolysaccharidosis type II^145–147^. Usually, variations that affect splicing occur in splicing regulatory sequences in the intronic and exonic regions^145^, and in this respect, it is important to note that even exonic variants that do not change the codon usage (i.e., same sense variants can also affect the splicing process^148^. Most of the reported NPC1 splicing variants are in the intronic regions, except for six variants located in exons 14, 17, 19, 22, and 24 (c.1553 G > A, c.2292 G > A c.2599c>T, c.2911 G > C, c.3422 T > G and c.3754 G > C)^145^. To this date, variants in the *NPC2* gene capable of affecting pre-mRNA splicing are less frequent; nonetheless, three intronic splicing variants have been reported in one study (IVS1 + 2 T > C; IVS2 + 5 G > A; IVS3 + 6 T > G). whilst an additional variant has been reported in the second one (IVS4 + 1 G > A)^142,149^.

The study of these variants could be particularly important for the development of future therapeutic strategies with the aim of rescuing mRNA splicing abnormalities, as has been done for several LSDs^150,151^. The most common splicing-therapeutics include the use of antisense oligonucleotide (AONs) and of modified U1 small nuclear RNAs (U1snRNA) targeting splicing regulatory sequences in the RNAs of interest^150^. Successful examples with U1snRNA have been explored in Fabry disease, Sanfilippo Syndrome, and Mucopolysaccharidosis^151–153^.

### Existence of complex alleles

Another factor that could partially explain phenotypic variability is the existence of complex alleles (CAs). CAs are defined as the presence of at least two genomic variants in the same parental allele. In most of the described cases, the functional effect of these variants is appreciated on the protein level. In addition, CAs can be challenging to detect due to the limitations of standard genetic screening methods. A few CAs that alter splicing have been reported and their effects could be predicted bioinformatically. For these cases, their functional analysis should be easier to determine than those that change the protein function. The reason is that variants in *cis* to native acceptor/donor splice sites could change their strength and modify the effect of other variants, which in turn could activate cryptic donor splice sites leading to pseudoexon inclusion or alter motifs of splicing enhancers/silencers and promote exon skipping^154,155^.

In this area, Bychkov and colleagues^145^ studied the impact of the frequent polymorphic variant c.2793 C > T, located in the donor splice site of NPC1 exon 18 on the rare synonymous variant c.2727 C > T, identified in two twins of 55 years old with adult-onset NPC. The variant leads to cryptic donor SS activation and frameshift deletion in the NPC1 exon 18. A minigene assay demonstrated that this exon shortening occurs only in the presence of the frequent polymorphic variant c.2793 C > T. Results of the transcript-specific qPCR showed that only the presence in the NPC1 exon 18 of both variants leads to a significant decrease in the WT transcript isoform^149^. However, since it is frequent among healthy controls and was not found in affected individuals, it is probably not pathogenetic^13^. Despite this conclusion, these results highlight the need to analyze raw sequencing data in cases where the identified variant could lead to cryptic SS activation. In fact, as observed in this study, any additional genetic variants nearby could affect the ratio of transcript isoforms and lead to a wrong genotype–phenotype correlation.

## Concluding remarks

LSDs, including NPC, are genetically and clinically heterogeneous. In these disorders, a broad phenotypic spectrum has been described, with differences in the age of onset of symptoms, rate of progression, disease severity, organs affected, effects on the CNS, and response to pharmacological treatments. This article has reviewed the phenotypic variation described in NPC and discussed their possible causes. The results of the studies performed so far lead to the general conclusion that NPC phenotypic expression can be modified by many factors, which include, but are probably not limited to, such as the level of residual function of the defective protein, genetic background, sex, environment, and splicing factors. This conclusion highlights the need to examine and categorize carefully the differences between individuals, which should be taken into account when designing hopefully effective treatments. Several other factors could influence NPC disease severity and have not been explored yet. Among them are epigenetic factors, the 3D architecture of the genome, the microbiome, and others (Fig. 2).

Up to this moment, NPC one-size-fits-all potential therapies that do not consider the wide phenotypic variation between patients suffering from this disease have been proposed, but as discussed in this review, their success has been quite limited. All these emerging data point toward the conclusion that biological differences among individuals should be considered when designing therapeutic approaches. If, in previous years, this goal could have seemed unreachable, with the current multi-omics advances, we should soon strive to be ushered into the precision medicine era for NPC and other LSDs.
